# Building healthcare capacity for neurodegenerative disease management in Nigeria: Challenges and opportunities

**DOI:** 10.1177/22799036251350957

**Published:** 2025-06-24

**Authors:** Elechi Kelechi Wisdom, Toluwalashe Soyemi, Shekoni Mayowa, Nwakaego Stephanie Ede, Elechi Ubalaeze Solomon, Chukwuagoziem Augustine Iloanusi, Chinonyelum Emmanuel Agbo, Obehi Suzan Idogen, Cletus Augustine Ikechukwu, Okechukwu Clinton Ifeanyi, Christiana Komeno Akpowowo, Farounbi Glory Oyebola, Chidinma Nndoumele, Lawal Olabisi Promise

**Affiliations:** 1 Integrated Biomedical Sciences, Graduate School of Biomedical Sciences, University of Texas Health Science Center, San Antonio, TX, USA; 2 Department of Medicine and Surgery, Lagos State University College of Medicine, Ikeja, Nigeria; 3 Department of Community Health and Primary Healthcare, Lagos State University College of Medicine, Ikeja, Nigeria; 4 Department of Physiotherapy, Faculty of Basic Medical Sciences, College of Medicine, University of Benin, Edo, Nigeria; 5 Department of Radiography and Radiological Sciences, Faculty of Health Science and Technology, University of Nigeria Enugu Campus, Nigeria; 6 Faculty of Clinical Sciences, University of Uyo, Nigeria; 7 Department of Clinical Pharmacy and Pharmacy Management, Faculty of Pharmaceutical Sciences, University of Nigeria, Nsukka, Nigeria; 8 Department of Optometry, Faculty of Life Sciences, University of Benin, Edo, Nigeria; 9 Department of Medical Laboratory Science, Ebonyi State University, Abakaliki, Nigeria; 10 Department of Medical Biochemistry, Faculty of Basic Medical Sciences, Delta State University, Abraka, Nigeria; 11 Department of Anatomy, Nnamdi Azikiwwe University Awka, Anambra State, Nigeria; 12 Department of Medical Laboratory Science, University of Benin, Edo State, Nigeria

**Keywords:** neurodegenerative diseases, brain health, public health, Nigeria, early detection, healthcare policy

## Abstract

Neurodegenerative diseases (NDs) such as Alzheimer’s disease (AD) and Parkinson’s disease (PD) are growing public health concerns worldwide, and their burden is particularly severe in low- and middle-income countries, including Nigeria. This perspective highlights the urgent need for tailored public health strategies to address the rising prevalence of these diseases, focusing on prevention, early detection, and management in the Nigerian context. With an aging population and limited healthcare infrastructure, Nigeria faces unique challenges in diagnosing and treating NDs. Cultural factors, such as stigma and misconceptions about cognitive decline, further complicate timely intervention. The paper explores the current public health strategies implemented to combat these diseases, including lifestyle modifications, national policy development, and public-private partnerships. It emphasizes the need for community-based programs, the integration of primary healthcare and neurology, and increased awareness to reduce the societal burden of NDs. Additionally, the manuscript discusses the essential role of training healthcare providers and the integration of traditional and modern medicine in enhancing care. It calls for a coordinated, culturally relevant approach to addressing the rising tide of neurodegenerative diseases in Nigeria, with recommendations for policy reform, healthcare system strengthening, and greater research investment.

## Introduction

Neurodegenerative diseases (NDs), which include Alzheimer’s disease (AD) and Parkinson’s disease (PD), are progressive disorders that cause the irreversible loss of neurons, significantly impacting brain function.^1^ As global populations age, the burden of these diseases has become a major public health concern. By 2050, the global prevalence of dementia is projected to rise from 50 million–115 million, with low- and middle-income countries, including Nigeria, being disproportionately affected.^2^ In Nigeria, the rise in aging populations, coupled with limited healthcare infrastructure, contributes to an increasing burden of NDs, particularly dementia, Alzheimer’s, and Parkinson’s disease.

This perspective focuses on the public health challenges and opportunities associated with the prevention and management of dementia, Alzheimer’s, and Parkinson’s disease in Nigeria. These three diseases are not only the most prevalent NDs worldwide, but they also present unique challenges in resource-limited settings. While dementia, especially Alzheimer’s, is widely recognized, Parkinson’s disease, often characterized by motor symptoms, is gaining attention due to its rising prevalence and associated burden on individuals and families. Justification for the focus on these diseases is rooted in their high prevalence and the significant impact they have on individuals, healthcare systems, and families in Nigeria. The rapid increase in dementia cases, with reports indicating a 400% rise over the past two decades, underscores the urgency of addressing this public health issue. However, cultural factors, such as the stigma surrounding cognitive decline, often impede early diagnosis and treatment, exacerbating the situation. Additionally, Nigeria’s healthcare system faces challenges in providing specialized care and adequate research infrastructure, leading to gaps in understanding the true extent of the problem. This paper explores existing public health strategies, identifies policy gaps, and discusses potential interventions that could alleviate the burden of these diseases in Nigeria. It emphasizes the need for culturally relevant, community-based programs that promote early detection, prevention, and improved healthcare delivery. The focus is on integrating evidence-based interventions that can be tailored to Nigeria’s unique cultural and socioeconomic context, highlighting the importance of a coordinated approach to promote brain health and prevent ND.

## Burden of neurodegenerative diseases in Nigeria

NDs, particularly Alzheimer’s disease (AD), Parkinson’s disease (PD), and Dementia, are emerging as significant public health challenges in Nigeria.^3^ The nation’s rapidly aging population, coupled with limited healthcare infrastructure, contributes to the increasing prevalence of these disorders.

### Alzheimer’s disease (AD)

#### Epidemiology

GBD 2019 estimates an age-standardized incidence rate (ASIR) of **75.3 per 100 000** (95 % UI 64.1–86.5) for Western Sub-Saharan Africa, the regional cluster that includes Nigeria; although the ASIR has shown a slight annual decline (EAPC –0.11 %), absolute incident cases continue to rise as the population ages.^4^

#### Risk-factor profile

Major Nigerian drivers include advanced age and the **APOE-ε4** allele, but modifiable factors such as mid-life hypertension, poorly controlled type-2 diabetes, chronic exposure to fine-particulate air pollution, and low educational attainment also increase risk.^5,6^

#### Socio-economic & cultural impact

Informal caregivers—predominantly women—supply >70% of unpaid care hours; stigma rooted in supernatural explanations delays formal diagnosis in ≈30 % of rural cases, compounding caregiver stress.^7^

#### Healthcare challenges

Fewer than 90 neurologists serve >200 million Nigerians; neuro-imaging and memory clinics are concentrated in six urban centers, and cholinesterase inhibitors face frequent stock-outs.^8^

### Parkinson’s disease (PD)

#### Epidemiology

According to GBD 2021, PD’s ASIR in Western Sub-Saharan Africa is **11.8 per 100 000** (95% UI 10.7–13.0); projections indicate a** ≈ 292 % rise in absolute cases by 2050**, the steepest regional increase worldwide.^9^

#### Risk-factor profile

Chronic pesticide exposure, well-water contamination by heavy metals, elevated total homocysteine, and repetitive head trauma are leading Nigerian risk factors; early-onset PD (<50 year) accounts for 13–15% of cases.^10,11^

#### Socio-economic & cultural impact

Monthly out-of-pocket levodopa costs can exceed 25% of household income, while early retirement or job loss amplifies financial toxicity.^12^

#### Healthcare challenges

Levodopa/carbidopa and dopamine agonists experience recurrent stock-outs; no dedicated movement-disorder clinics exist outside Lagos and Abuja, and deep-brain-stimulation is unavailable in public hospitals.^11,13^

### All-Cause dementia (Non-AD, Non-PD)

#### Epidemiology

GBD 2019 reports an ASIR of 73–76 per 100,000 for “Alzheimer’s disease and other dementias” in Western Sub-Saharan Africa—virtually identical to AD because regional GBD aggregates them—implying that non-Alzheimer dementias contribute substantially to the overall burden.^4^ A 2019 Nigerian systematic review found a pooled crude prevalence of 4.9% (95% CI 3.0–6.9) among adults ≥60 year, with significantly higher prevalence in women (6.7%) than men (3.1%).^14^

#### Risk-factor profile

Low education, undernutrition (BMI <18.5 kg m^−2^), hypertension, diabetes, obesity, chronic hearing loss, and social isolation all elevate risk in Nigerian cohorts.^5,6,15^

#### Socio-economic & cultural impact

Caregiver burden is characterized by high levels of stress, physical exhaustion, and out-of-pocket expenditures; dementia is still widely misinterpreted as witchcraft, leading to violence and abandonment of elderly women.^7^

#### Healthcare challenges

Diagnostic services are sparse, memory clinics are limited to a handful of teaching hospitals, and no national dementia plan exists. A recent hospital audit highlighted delayed presentation (>18 months after symptom onset) and limited follow-up due to travel costs^16^ (Table 1).

**Table 1.** Key global burden of disease (GBD) incidence metrics for Nigeria’s region (Western SSA).

Disorder	Metric (per 100 000)	Year	Source
Alzheimer’s disease	ASIR 75.3 (64.1–86.5)	2019	GBD 2019^4^
Parkinson’s disease	ASIR 11.8 (10.7–13.0)	2021	GBD 2021^9^
All-cause dementia	ASIR ≈ 75 (64–87)*	2019	GBD 2019^4^

* GBD aggregates “Alzheimer’s disease and other dementias”; thus, the dementia figure approximates non-AD dementias when AD is subtracted.

## Current public health strategies

Addressing NDs such as Alzheimer’s disease (AD) and Parkinson’s disease (PD) has become a significant public health priority in Nigeria. The country’s rapidly aging population, coupled with urbanization and lifestyle changes, has led to an increased burden of these conditions. Tackling dementia and other NDs in Nigeria necessitates a comprehensive approach that integrates lifestyle modifications, public awareness, and strategic collaborations. Recent studies have shown that certain lifestyle behaviors are linked to a reduced risk of NDs, prompting a shift in public health strategies.^17^ Notably, unmanaged cardiometabolic health, sedentary lifestyles, poor dietary habits, obesity, smoking, and excessive alcohol consumption are modifiable risk factors for NDs such as dementia.^18–21^ Targeting these factors through public health interventions is essential to mitigate the growing burden of NDs in the country. In response, Nigeria has implemented several public health strategies aimed at prevention, early detection, and management of NDs.

### National policy development

Recognizing the growing challenge posed by NDs, the Nigerian government has developed national policies to guide public health responses. These policies align with global frameworks, such as the World Health Organization’s Global Action Plan on the Public Health Response to Dementia (2017–2025), which emphasizes dementia as a public health priority and advocates for risk reduction strategies, early diagnosis, and support for caregivers.^22^ While specific details of Nigeria’s national plan are not extensively documented, efforts are underway to integrate these global recommendations into local contexts.

### Public awareness and education

Increasing public awareness about NDs is a cornerstone of Nigeria’s public health strategy. Organizations like the Alzheimer’s Disease Association of Nigeria (ADAN) have been instrumental in this regard. Established in 1999, ADAN has initiated monthly dementia awareness programs, established early dementia diagnosis clinics within primary healthcare settings, and conducts regular public health education meetings. These initiatives aim to dispel myths, reduce stigma, and encourage early consultation with healthcare professionals.^23^

### Lifestyle modification initiatives

Addressing modifiable risk factors is central to preventing and managing NDs. Public health campaigns in Nigeria focus on promoting:

- **Tobacco Cessation:** Programs aimed at reducing tobacco use, which is associated with an increased risk of dementia.
- **Alcohol Moderation:** Initiatives to decrease hazardous alcohol consumption, known to contribute to cognitive decline.
- **Healthy Diet Promotion:** Encouraging diets rich in fruits, vegetables, and fish, which have been linked to a lower risk of AD.^24^
- **Physical Activity:** Promoting regular exercise to enhance vascular health and reduce dementia risk.
- **Management of Comorbidities:** Ensuring effective control of hypertension, diabetes, and dyslipidemia, which are linked to increased dementia risk.

These lifestyle modifications are disseminated through community workshops, media campaigns, and collaborations with healthcare providers.

### Capacity building and training

Enhancing the skills of healthcare professionals is vital for effective ND management. Training programs focus on early detection, diagnosis, and management of NDs, equipping professionals with the necessary tools to address the unique challenges presented by these conditions. Additionally, research initiatives are being undertaken to understand the genetic and environmental factors contributing to NDs in the Nigerian context.

### Support for caregivers

Recognizing the essential role of caregivers, public health strategies include support systems such as counseling, respite care, and support groups. These services aim to alleviate the emotional and physical burden on caregivers, ensuring they have the resources and support needed to care for individuals with NDs.

### Collaboration with non-governmental organizations (NGOs)

NGOs play a pivotal role in Nigeria’s public health strategy for NDs. Collaborations with organizations like ADAN have led to the establishment of support groups, educational seminars, and community outreach programs. These partnerships enhance resource mobilization and extend the reach of public health initiatives.

### Research and surveillance

Ongoing research is crucial for understanding the prevalence, risk factors, and effective interventions for NDs in Nigeria. Studies have provided insights into the incidence of dementia and AD, highlighting dietary factors such as fat intake and fish consumption near the time of onset as significant risk factors.^25,26^ Continuous surveillance informs policy decisions and the allocation of resources to areas with the greatest need.

In July 2018, during a meeting at the World Health Organization (WHO) Headquarters in Geneva, several interventions were recommended to potentially reduce the future risk of dementia and cognitive decline.^27^ These included interventions for tobacco cessation, measures to reduce or eliminate hazardous drinking, the promotion of a healthy and balanced diet, as well as the management of hypertension, diabetes, and dyslipidemia in adults. These recommendations were incorporated into the Global Action Plan on the Public Health Response to Dementia (2017–2025), the Comprehensive Mental Health Action Plan (2013–2020), and the Global Action Plan for the Prevention and Control of Noncommunicable Diseases (2013–2020). These plans emphasize the importance of adopting a holistic approach to dementia prevention and the management of other cognitive diseases, particularly by addressing lifestyle factors from an early age.

**Figure 1.**
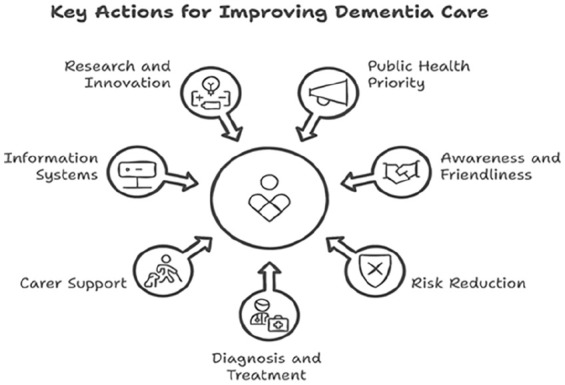
Key actions for improving dementia care.

The WHO’s Global Action Plan on the Public Health Response to Dementia sets out to create a world where dementia can be prevented, and individuals living with dementia, as well as their caregivers, receive the support and care they need to live with dignity, respect, autonomy, and equality. The plan outlines seven key action areas, which include Figure 1:

1. Dementia as a public health priority
2. Dementia awareness and friendliness
3. Dementia risk reduction
4. Diagnosis, treatment, care, and support for dementia
5. Support for dementia carers
6. Use of information systems
7. Research and innovation

Among these, Action Area three focuses on dementia risk reduction by reducing exposure to harmful risk factors such as tobacco use, obesity, diabetes, and hypertension throughout an individual’s life. This requires enhancing the capacity of health and social care professionals to offer evidence-based interventions, educate, and proactively manage modifiable risk factors to reduce or delay the onset of dementia. The goal is to achieve a 25% reduction in premature mortality from dementia by 2025.

In Nigeria, non-governmental organizations (NGOs) play a critical role in dementia prevention by raising awareness and organizing seminars on dementia-related topics. They contribute to the broader public health effort by fostering a better understanding and engagement with dementia risk reduction strategies. Public awareness and education campaigns are crucial because current public knowledge of dementia risk reduction remains low. Many individuals are unaware that dementia risk reduction is possible, and fewer know how to achieve it. For instance, the Dementia Friends program was launched in Nigeria on January 28, 2016, aiming to change the way the nation thinks, acts, and talks about dementia.^28^ Within a few months, over 21 Dementia Friends Champions were established across 19 of the 36 states. Each Champion was tasked with creating 100 Dementia Friends, delivering information sessions in schools, hospitals, places of worship, and other community venues. This grassroots approach has significantly increased public awareness and understanding of dementia. Furthermore, the Africa-FINGERS project represents a significant initiative in Nigeria, focusing on culturally informed, multimodal interventions to promote healthy brain aging. This project involves randomized control trials in urban and rural sites, enrolling 600 at-risk adults aged 50 and above. The goal is to develop and test strategies to reduce the risk of Alzheimer’s Disease and Related Dementias (ADRD) in the African context. The Africa-FINGERS initiative highlights the potential for scalable and sustainable interventions, poised to transform dementia risk reduction efforts across the continent.^29^

In summary, addressing dementia and other NDs in Nigeria requires multifaceted public health strategies, including lifestyle modifications, widespread awareness campaigns, and collaborations with NGOs. As the WHO’s Global Action Plan progresses toward its 2025 targets, Nigeria is working to implement these initiatives at local and national levels to reduce the prevalence and impact of dementia and enhance the well-being of affected individuals and their caregivers.

## Challenges in promoting brain health

Promoting brain health in Nigeria is hindered by several interrelated challenges, including inadequate healthcare infrastructure, cultural misconceptions, limited access to specialized care, and insufficient research funding.

### Healthcare infrastructure and resource limitations

Nigeria’s healthcare infrastructure is underdeveloped, particularly in rural areas where facilities often lack essential diagnostic and treatment equipment for NDs. The country has approximately 80 registered neurologists serving a population exceeding 200 million, highlighting a significant shortage of specialized professionals.^30^

### Cultural perceptions and stigma

Cultural beliefs in Nigeria frequently attribute symptoms of NDs to supernatural causes, leading to delayed medical intervention. This stigma contributes to social isolation and discourages individuals from seeking healthcare, often opting for traditional healers instead.^31,32^

### Access to care and diagnostic services

Many Nigerians, especially in rural regions, face challenges accessing specialized healthcare.^32–34^ High costs of diagnostic tests and long-term care make treatment unaffordable for many, while limited awareness of early disease symptoms contributes to delayed diagnoses and interventions.

### Funding and research gaps

**Figure 2.**
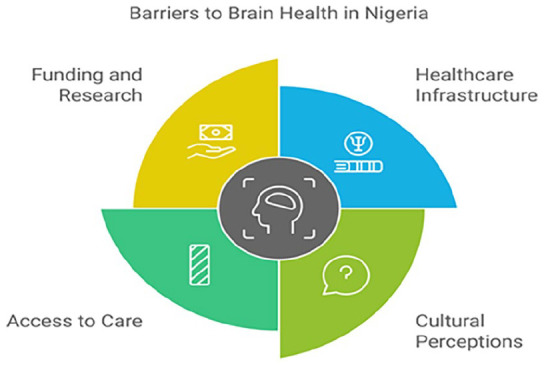
Barriers to brain health in Nigeria.

Insufficient funding for ND research hampers the development of localized solutions.^35^ A lack of comprehensive epidemiological data impedes effective policy planning, and the emigration of skilled researchers, coupled with limited global collaborations, further restricts advancements in the field (Figure 2).

## Proposed public health interventions

### Community-based programs for early detection and prevention

Implementing community-based awareness programs is a crucial strategy for raising public awareness and preventing NDs. These initiatives should focus on promoting brain health across all life stages by addressing key determinants such as physical health, environmental factors, safety, education, social connections, and access to quality services. Encouraging the adoption of lifestyle practices that mitigate NDs risk factors and enhance protective factors can significantly improve brain adaptability and resilience. Such programs are essential for overcoming cultural perceptions and stigma associated with NDs. For instance, the Dementia Friends program, launched in Nigeria in early 2016, has successfully increased awareness, with over 80,000 individuals participating across 19 states.^33^

### Strengthening healthcare systems and workforce training

NDs Contribute Significantly To Morbidity and disability, necessitating a comprehensive, patient-centered healthcare approach. Ensuring equitable access to competent care, integrating services at all healthcare levels, and providing essential medications and diagnostics are crucial for achieving universal health coverage and meeting Sustainable Development Goals. A robust healthcare system should facilitate timely diagnosis, treatment, and long-term care, thereby enhancing quality of life, preventing complications, reducing hospitalizations, and minimizing premature mortality. Coordinated national public health efforts are required to support professionals and caregivers in effectively managing NDs and alleviating their societal and economic burdens. A case study from Benue State demonstrated the feasibility of rapidly scaling up mental health services in primary care through public-private partnerships, integrating mental health into primary care in alignment with national policies and the WHO’s mhGAP-IG.^34^

### Public-private partnerships in brain health

Developing evidence-based policies to address NDs necessitates systematic research, which is essential for understanding service delivery, care strategies, treatment options, fostering innovation, and ensuring equitable access to health technologies. Effective research requires enhanced coordination among stakeholders, the establishment of public-private partnerships, and optimal resource utilization. Therefore, a national public health initiative aimed at preventing NDs should include an inclusive public-private partnership framework to boost research funding and governance. Encouraging public-private partnerships can promote novel drug development and expedite the availability of treatments for patients with brain disorders in Nigeria.^30^

### Integration of traditional and modern medicine approaches

**Figure 3.**
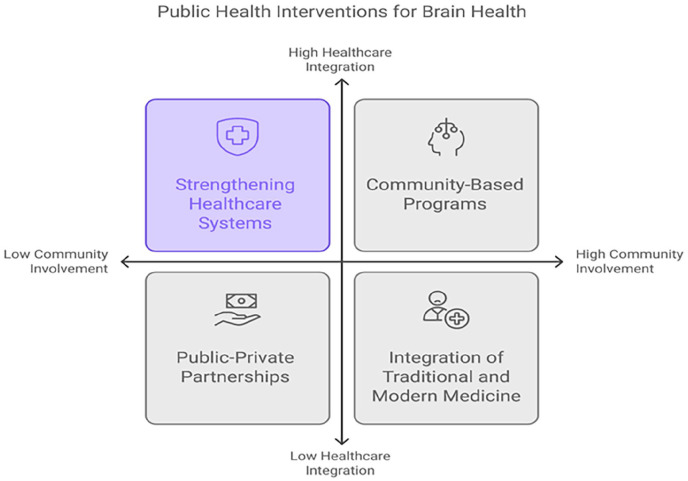
Public health interventions for brain health.

Incorporating both traditional and modern medicine is crucial for a comprehensive approach to ND prevention and treatment in Nigeria. Traditional medicine, deeply rooted in Nigerian communities, offers accessible treatments, though they often lack scientific validation. Structured collaboration between traditional healers and modern medical practitioners can bridge this gap by establishing guidelines for safe herbal treatments and generating clinical evidence through scientific research. The World Health Organization advocates for integrating traditional medicine with evidence-based modern healthcare.^36^ Public education campaigns should also be implemented to raise awareness about the benefits and limitations of both traditional and modern treatments, reducing stigma. Ultimately, a balanced approach will enhance the accessibility, affordability, and cultural relevance of ND care in Nigeria, ensuring comprehensive and inclusive healthcare (Figure 3).

## Early detection and screening programs

### Community-based screening initiatives

Implementing community-based screening programs enhances accessibility to early diagnoses and treatments for NDs like Parkinson’s and dementia.^37^ These initiatives can reduce diagnostic barriers, as evidenced by studies highlighting their effectiveness. Community health workers are essential in promoting brain health through educational programs, conducting screenings, and supporting patients and caregivers. However, challenges such as inconsistent funding and resource distribution can impact sustainability, underscoring the need to address these issues to improve cognitive impairment detection and overall brain health.

### Mobile health clinics and community health worker involvement

Deploying mobile health clinics provides primary healthcare access, bringing services directly to various communities. This approach encourages participation in health checks, including screenings for neurodegenerative conditions, benefiting individuals who might otherwise forgo assessments due to financial constraints, low awareness, or misconceptions. Integrating community health workers with mobile clinics ensures early detection services are available across urban and rural areas, facilitating timely diagnoses and preventive care. Studies have shown that mobile health clinics play a vital role in community healthcare, serving as an essential first point of contact for primary care in rural areas.

### Integration with primary healthcare

Integrating neurological assessments into primary healthcare makes neurological health a core component of overall care. Incorporating these evaluations into routine check-ups enables early detection and intervention. Research indicates that such integration improves outcomes.^38^ However, a shortage of specialists and resources poses challenges in developing countries. Strengthening primary healthcare with neurological evaluations can enhance early diagnosis, treatment, and management of NDs, reducing long-term burdens. Integrating brain disorder assessments with general healthcare has been shown to improve outcomes, though resource limitations may pose challenges.^39,40^

### Training for healthcare providers in early detection

Early ND detection depends not only on healthcare workers’ availability but also on their competence in neurohealthcare. Many providers lack specialized neurology training, worsened by workforce shortages in Africa. A study in Southeastern Nigeria found insufficient knowledge of Alzheimer’s, leading to missed diagnoses.^41^ Basic neurohealth training is essential to improve recognition and assessment of ND conditions. Education programs enhance healthcare workers’ ability to identify and manage diseases, making expanded neurotraining crucial for early detection and intervention.

## Future directions in brain health

### Emerging research and innovations

Over the past two decades, local and international initiatives have been launched to address NDs like Alzheimer’s, Parkinson’s, Huntington’s, Mad Cow disease, Frontotemporal dementia, and Amyotrophic lateral sclerosis. These conditions are caused by the misfolding and aggregation of proteins, impairing neuron function.^42^ Recent advancements include inhibitors targeting these amyloidogenic processes. Research has also highlighted the role of the “Exposome,” which encompasses an individual’s total environmental exposures, including lifestyle, diet, pollutants, and chemicals.^43^ This concept explains the incomplete heritability of NDs, with genetic factors accounting for only a portion of the risk. Environmental exposures are now recognized as playing a significant role in the development and progression of these diseases.

### Global collaborations and best practices

The global burden of neurological disorders is rising, particularly in low-resource settings, prompting an increase in neuroscience publications from countries like South Africa, Egypt, and Nigeria. Despite challenges, Africa is working to catch up with the scientific advancements of other regions. Neuroscience is entering a new era of collaboration, with large global projects making a significant impact on medical science, economics, and society. Initiatives such as the US BRAIN initiative, the EU’s Human Brain Project, Japan’s Brain/MINDS, and others from Korea, China, Canada, and Australia are driving innovation and international cooperation in brain research.^44^

### Long-term goals and sustainability

ND diseases are related to aging, genetics, behavior, environmental factors, and infections. Environmental exposure to heavy metals, pollution, poor diet, microorganisms, and toxins like pesticides contribute to cognitive impairment, motor abnormalities, mental disturbance, and sensory deficiencies. Neurotransmitter processes in the nervous system are well-regulated, and toxic pollutants can truncate these functions. Preventive measures, such as promoting healthy lifestyles and early detection, are crucial for long-term sustainability. Prospective ND disease management includes both pharmacological and non-pharmacological approaches, majoring in halting disease progression, such as using heat shock protein 104 (Hsp104), immunomodulators, autophagy, and other emerging therapies.^45,46^

## Conclusion

Promoting brain health and preventing NDs in Nigeria requires a comprehensive public health approach that accommodates a broader range of conditions. The growing burden due to an aging population and increased incidence of both dementia and Parkinson’s disease necessitates immediate attention. By integrating evidence-based interventions and addressing the cultural challenges faced by affected individuals, Nigeria can enhance brain health outcomes significantly. Future research should prioritize context-specific solutions that account for the multifaceted nature of ND diseases and foster collaborations to address the complexities involved in managing these conditions.
